# Dexamethasone modified by gamma-irradiation as a novel anticancer drug in human non-small cell lung cancer

**DOI:** 10.1371/journal.pone.0194341

**Published:** 2018-04-04

**Authors:** Eun-Hee Lee, Chul Hong Park, Hyo Jin Choi, Remigius Ambrose Kawala, Hyoung-Woo Bai, Byung Yeoup Chung

**Affiliations:** 1 Research Division for Biotechnology, Advanced Radiation Technology Institute (ARTI), Korea Atomic Energy Insitute (KAERI), 29 Geumgu-gil, Jeongeup-si, Jeollabuk-do, Republic of Korea; 2 Daegu-Gyeongbuk Medical Innovation Foundation, Medical Device Development Center, Daegu, Republic of Korea; 3 Radiation Biotechnology and Applied Radioisotope Science, University of Science and Technology (UST), Daejeon, Republic of Korea; Northwestern University Feinberg School of Medicine, UNITED STATES

## Abstract

Dexamethasone (Dex) is widely used in the management of leukemia and lymphoma. While Dex is commonly used for hematological malignancies, the effects of Dex in solid cancer cells remain controversial. To develop a more effective anticancer drug for solid cancers, Dex was modified by ionizing radiation and the anticancer activity of ionizing-radiation-irradiated Dex (Dex-IR) was investigated in human lung cancer cells. Using the MTT assay, the proliferation of non-small cell lung cancer cells was significantly inhibited after treatment with Dex-IR compared with Dex. Furthermore, Dex-IR induced apoptotic cell death and cell cycle arrest of H1650 human lung cancer cells. The invasiveness of H1650 cells was significantly reduced and the matrix metalloproteinase activity was strongly suppressed. These results indicate that Dex-IR acts as a tumor suppressor by both inducing apoptosis and arresting the cell cycle. Although the structure of Dex-IR remains to be determined, our results suggest it may be useful as a novel anticancer agent for the treatment of solid cancers.

## Introduction

Glucocorticoids (GCs) inhibit various cellular, molecular, and physiological networks in organisms and are used to treat autoimmune and lymphoproliferative disorders [1]. In addition to using GCs as an adjuvant, GCs also alleviate the side effects of chemotherapy and radiotherapy for many cancer types [2]. Dexamethasone (Dex) is one of the most widely used synthetic GCs and used in combination with chemotherapy for several solid tumors, such as prostate and lung cancer [3–6]. However, despite the beneficial effects of Dex, it can potentially induce the steroids-associated with variety side effects, promote tumor proliferation or metastasis [7]. Some interesting approaches have been used to efficiently develop the GCs via structural modification using additional functional groups [8] or aliphatic or aromatic linker molecules [9] to generate improved innovative drugs. However, little is known of the use of ionizing radiation to produce favorable changes through the rearrangement of chemical bonds.

Recently, several research groups have reported that ionizing radiation has the potential to develop new compounds with enhanced bioactivity [10, 11]. Previously, we demonstrated that rotenoisin B, a gamma-irradiated rotenone compound, inhibited the proliferation of hepatic cancer cells [12]. To date, no reports have discussed the effects of γ-irradiation on Dex. Previous studies have clearly suggested that the exposure of Dex to ionizing radiation might result in effective novel drugs that target solid cancers. Therefore, we modified Dex using radiation to develop new candidate drugs. We hypothesized that the Dex modified by ionizing radiation (Dex-IR) would be a more powerful anticancer drug for non-hematologic malignancies and speculated that Dex-IR might induce cell death and inhibit the invasion of non-small cell lung cancer Cells (NSCLC).

## Materials and methods

### Cell lines, chemicals and reagents

Human NSCLC cell lines H1650, A549 and H1299 were obtained from the American Type Culture Collection (ATCC, Manassas, VA, USA). All cells were grown in RPMI-1640 medium supplemented with 10% fetal bovine serum (FBS) at 37°C in a 5% CO_2_ atmosphere. Dex, doxorubicin (DOXO) and nocodazole were purchased from Sigma-Aldrich (St. Louis, MO, USA). Immunoblot analyses were conducted using the following antibodies: anti-Caspase-3, anti-cleaved Caspase-3, anti-poly(ADP-ribose) polymerase (PARP), anti-cleaved PARP, anti-Cyclin A2, anti-Cyclin D1, anti-Cyclin B1, anti-phospho-Rb (Ser807/811) and anti-GAPDH (Cell Signaling Technology, Beverly, MA, USA).

### Gamma irradiation and LC-MS

One gram of stock solution of Dex dissolved in 1 L of methanol was irradiated by ionizing radiation with γ-rays in a ^60^Co γ-chamber for 2 h for a total dose of 20 kGy with a dose rate of 10 kGy h^-1^. The samples were irradiated at room temperature (RT) in air using a ^60^Co irradiator (MDS Nordion, Ottawa, ON, Canada). After evaporation *in vacuo*, the samples were analyzed by liquid chromatography tandem mass spectrometry (LC-MS/MS) or high-performance liquid chromatography (HPLC) to determine the novel fraction of γ-irradiated Dex components by comparing the chromatogram with that of the original Dex. The LC-MS/MS (Agilent Technologies, Palo Alto, CA, USA) analysis was performed in a YMC-Pack ODS A-302 column (4.6 mm i.d. × 150 mm; YMC, Kyoto, Japan) and the solvent system consisted of a linear gradient that started with 0.1% HCOOH (detection: UV 254 nm; flow rate: 1.0 mL/min; at 40°C), was increased to 50% (v/v) MeCN in 0.1% HCOOH/H_2_O over 20 min, and then increased to 100% MeCN over 20 min [13]. At the end of a run, an additional 15 min was used to allow equilibration of the column. This γ-irradiated Dex was named Dex-IR and five main peaks were detected.

### MTT assay of cell viability

Stock solutions were prepared with dimethyl sulfoxide (DMSO). The final DMSO concentration in the culture medium was below 1%. H1650 cells were seeded in 96-well flat-bottom plates at a density of 2 × 10^4^ cells/well and treated with various concentrations of each compound in fresh medium. After 24-h incubation, MTT (5 mg/mL in water; 20 μL/well) was added to the wells and incubated at 37°C for 4 h. The supernatant was removed and DMSO (100 μL/well) was added to dissolve the formazan produced. After shaking the plates, the absorbance of the wells was determined with a microplate reader (Tecan, Männedorf, Switzerland) at 570 nm.

### Annexin V apoptotic assay

H1650 cells were seeded in 6-well plates and grown for 24 h. Then, 72 h after treatment with 100 ug/mL of Dex, Dex-IR, or control DMSO (1%), the cells were harvested by trypsinization for analysis. Cell viability was determined with a Muse Cell Analyzer (Merck Millipore, Billerica, MA, USA) with the Muse Annexin V & Dead Cell kit, according to the manufacturer’s instructions. Briefly, the harvested cells were stained with the Annexin V/propidium iodide (PI) mixture in binding buffer from the kit for 20 min at RT in the dark. For each sample, 20,000 events were collected. The percentages of apoptotic and necrotic cells were determined using Muse 1.1.2 analysis software. Early apoptotic cells (Annexin V-FITC+/PI−) can be distinguished from late apoptotic cells (Annexin V-FITC+/PI+). Live cells are Annexin V and PI-double-negative (Annexin V-FITC−/PI−), whereas necrotic cells are PI-single-positive (Annexin V− / PI+).

### TdT-mediated dUTP nick end-labeling (TUNEL) assay

Cells were seeded onto coverslips and treated with drugs for 12 h. The TUNEL assay was performed with the DeadEnd Colorimetric TUNEL system (Promega, Madison, WI, USA), according to the manufacturer’s instructions [14]. After counterstaining the samples with 4,6-diamidino-2-phenylindole (DAPI), images were analyzed directly with an Olympus IX71 fluorescence microscope (Olympus, Tokyo, Japan).

### Immunoblot analysis

The treated cells were lysed using radioimmunoprecipitation lysis buffer (Rockland, Limerick, PA, USA), centrifuged, and the supernatant was collected. The protein concentration was determined using the BCA assay, according to the manufacturer’s instructions (Thermo Fisher Scientific, Seoul, Korea). Cell lysates containing equal amounts of protein (50 ug) were prepared and separated by 10–15% sodium dodecyl sulfate-polyacrylamide gel electrophoresis and transferred to PVDF membranes (Merck Millipore, Billerica, MA, USA). After blocking with Tris-buffered saline containing 0.1% Tween-20 and 5% skim milk, the membrane probed with a primary antibody was shaken at 4°C overnight and then incubated with the secondary antibody for 1 h at RT. The protein bands were detected with the ECL system (Thermo Fisher Scientific, Seoul, Korea).

### Cell cycle assay

After treatment with 100 ug/mL Dex, Dex-IR, or control DMSO (1%), the cells were harvested and fixed with 70% cold ethanol at –20°C for 24 h. The fixed cells were washed and 150 μL of Muse™ Cell cycle reagent was added. The cells were incubated at RT for 30 min in the dark. For each sample, 10,000 events were collected. The samples were analyzed using the Muse Cell Analyzer.

### Matrigel invasion assay

A Matrigel invasion assay was conducted with BioCoat Matrigel invasion chambers (Becton Dickinson Labware, Franklin Lakes, NJ, USA), according to the manufacturer’s protocol. Briefly, 1 × 10^5^ cells were resuspended in 100 μL of serum-free medium and placed in the upper compartment of a Transwell invasion chamber (8 μm pores). To the lower compartment of the chamber, 800 μL of RPMI with 10% FBS containing drugs was added as a chemoattractant. After incubation for 24 h at 37°C in 5% CO_2_, any cells that had not invaded the filter were wiped away with a cotton swab, while the cells that had invaded the lower surface of the filter were fixed with 100% methanol. The cells were then stained with hematoxylin and eosin (Sigma-Aldrich) and counted in five randomly selected microscopic fields (400×) per filter.

### Gelatin zymography

Zymography was performed using 7.5% (w/v) polyacrylamide gels containing 4 mg/mL gelatin, as previously described [15]. Briefly, at 70–80% confluence, the medium containing FBS was removed, and the cells were allowed to grow in FBS-free medium treated with drugs. After 24 h, collected samples were concentrated and mixed with sample buffer (125 mM Tri-HCl pH 6.8, 4% SDS, 20% glycerol, and 0.01% bromophenol blue) and applied directly to a gel. After removing the SDS from the gel by incubation in washing buffer (2.5% Triton X-100, 50 mM Tris-HCl pH 7.5, 5 mM CaCl_2_, and 1 μM ZnCl_2_) for 1 h, the gels were incubated at 37°C for 18 h in incubation buffer (1% Triton X-100, 50 mM Tris-HCl pH 7.5, 5 mM CaCl_2_, and 1 μM ZnCl_2_). The gels were stained for 1 h in Coomassie Brilliant Blue G250 and destained in the same solution without dye for 2 h. Any gelatinolytic activity was evident as a clear band against the blue background of the stained gelatin.

### Quantitative real time-PCR

Total RNA was harvested from H1650 cells treated with drugs using TRIzol reagent (Molecular Research Center, Cincinnati, OH, USA), and 2 ug of RNA was reverse transcribed with a GoScript complementary DNA (cDNA) Synthesis kit (Promega) to synthesize cDNA. Reverse transcription-polymerase chain reaction (RT-PCR) was performed using Emerald Amp PCR Master Mix (Takara, Otsu, Japan), according to the kit instructions. The resulting cDNA was amplified with the following primers: β-actin, 5ʹ-GTGGGGCGCCCCAGGCACCA-3ʹ and 5ʹ-CTCCTTAATGTCACGCACGAT-3ʹ; MMP2, 5ʹ-ACATCAAGGGCATTCAGGAG-3ʹ and 5ʹ- TGAACCGGTCCTTGAAGAAG-3ʹ; MMP9, 5ʹ-GAGTTCCCGGAGTGAGTTGA-3ʹ and 5ʹ-GGGATTTACATGGCACTGCC-3ʹ; integrin α2, 5ʹ- ACATTCAGGCATACCCATTT-3ʹ and 5ʹ-TGGCTACAGTTTCAGTCTTT-3ʹ; and integrin α5, 5ʹ- GTGGGCCAACAAAGAACACT-3ʹ and 5ʹ-TGAGTTCTGATTCCCCTTGG-3ʹ. Quantitative real-time PCR was performed using a Bio-Rad CFX-96 real-time PCR system (Bio-Rad, Hercules, CA, USA) and SYBR Green PCR Mater Mix (Takara, Dalian, China). The Ct value was analyzed using the instruments’ software. The relative transcription levels were estimated using the 2–^ΔΔCt^ method. The qPCR results for each gene and sample were studied in triplicate.

### Statistical analysis

Statistical differences were evaluated using the Student’s *t*-test. The results were considered statistically significant when *P*-values were < 0.05. All experiments were performed at least five times independently.

## Results

### Dex-IR suppresses the proliferation of NSCLC cells

**Fig 1:**
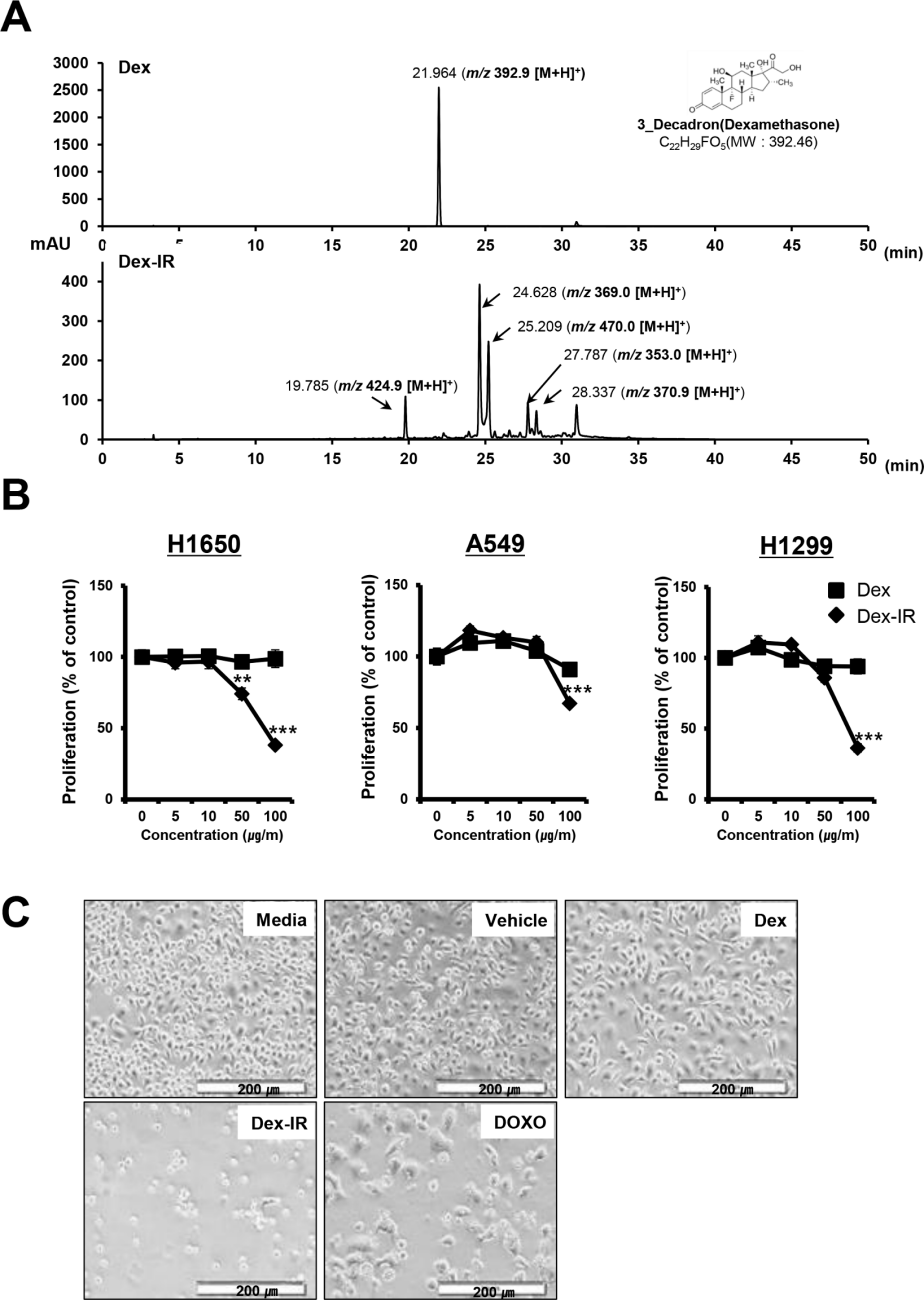
Ionizing-radiation-irradiated Dex (Dex-IR) inhibits the proliferation of non-small cell lung cancer (NSCLC) cells. (A) The chromatograms of Dex (top) and the fraction of crude extracts with Dex-IR (bottom). The chromatography conditions are given in the Materials and Methods. The arrows indicate the retention time of each peak. (B) Lung cancer cell lines were treated with increasing concentrations of Dex and Dex-IR for 24 h. The effects of Dex-IR at the indicated concentration on the viability of lung cancer cells were determined using the MTT assay and were compared with those of Dex-treated cells. Data are presented as the mean ± standard error of the mean (SEM) of three independent experiments (*P < 0.05). (C) H1650 cells were treated with vehicle (1% DMSO), Dex (100 ug/mL), Dex-IR (100 ug/mL), or doxorubicin (DOXO; 1 μM) for 72 h. DOXO was used as a positive control. Cells were observed using phase-contrast microscopy (40× magnification). The scale bar is 200 μm.

To detect the novel Dex-IR compounds, sample solutions containing Dex and Dex-IR in methanol were monitored by LC-MS analysis. Five main peaks were seen with Dex-IR (Fig 1A). Using Dex as a standard, all of the Dex-IR peaks were new, indicating that the Dex was completely altered by γ-irradiation.

Dex inhibits the proliferation of NSCLC cells, but has minimal cytotoxic effects [16]. The reported IC_50_ of Dex for A549 and H1650 cells exceeded 500 μmol/L (at 196 mg/mL) and Dex had no effect on A549 cell proliferation at low doses (0.1 and 1 μmol/L). To evaluate whether Dex-IR has an anti-cancer effect on NSCLC, the MTT assay was used to assess cell cytotoxicity. NSCLC cells were treated with various concentrations of Dex-IR, as indicated in Fig 1B. Compared with Dex, the proliferation of Dex-IR-treated NSCLC cells was significantly inhibited at a concentration of 100 ug/mL for 24 h. Both H1650 (38.2%) and H1299 (36.3%) cells were more sensitive to the Dex-IR treatment than A549 cells at 100 ug/mL. Similarly, changes in cell morphology and confluence were observed with phase contrast microscopy (Fig 1C). Compared with untreated or DMSO-treated cells, more Dex-IR-treated cells floated, indicating reduced adherence.

These results suggest that while Dex has no effect on the proliferation of NSCLC cells at low concentrations, Dex-IR inhibited the proliferation of these lung cancer cells.

### Dex-IR induces apoptotic cell death

**Fig 2:**
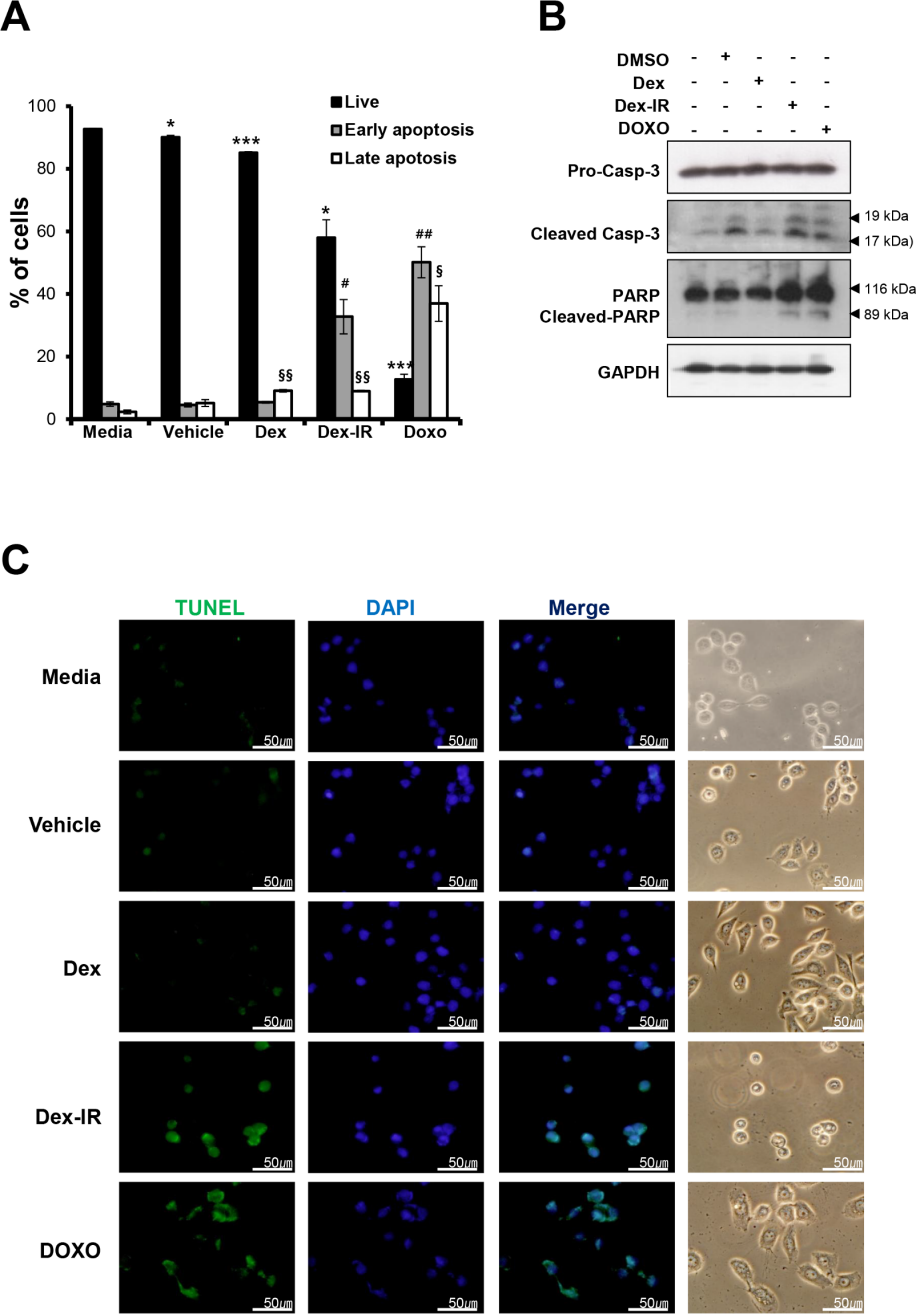
Increased apoptotic cell death induced by Dex-IR in H1650 lung cancer cells. (A) Annexin V/propidium iodide double staining analysis of apoptosis in H1650 cells. H1650 cells were treated with Dex, Dex-IR, or DOXO as described in Fig 1 for 72 h. The bar graph shows the percentages of dead, living, early-apoptotic, and late-apoptotic cells according to treatment. Data are presented as the mean ± SEM of three independent experiments (*P < 0.05 *vs*. live cells in the medium; ^#^*P* < 0.05 *vs*. early apoptotic cells in the medium; and ^§^*P* < 0.05 *vs*. late apoptotic cells in the medium). (B) After treating H1650 cells with the same doses of the indicated drugs as described above for 24 h, the proteins were analyzed by Immunoblotting with antibodies against pro-Casp-3, cleaved Casp-3, PARP, and cleaved PARP. GAPDH was used to normalize the protein contents. (C) Dex-IR-induced apoptotic cells were detected by TUNEL assay (400× magnification). H1650 cells were treated with the drugs as described in Fig 1 for 12 h.

To assess the Dex-IR-induced apoptosis of lung cancer cells, Annexin V-fluorescein isothiocyanate/PI was used to stain H1650 cells treated with Dex, Dex-IR, or DOXO for 72 h. Although late apoptotic cells were increased among the cells treated with both Dex and Dex-IR compared with the control, the percentage of early apoptotic cells was increased significantly after treatment with Dex-IR (58%) (Fig 2A). To determine whether apoptotic signaling molecules are involved in the Dex-IR-induced apoptotic cell death, immunoblotting analysis was performed. As shown in Fig 2B, the expression of certain apoptotic marker proteins, including cleaved Casp-3, and cleaved PARP, was detected after treatment with Dex-IR and DOXO as a positive control. To further elucidate whether Dex-IR-induced DNA fragmentation was a result of apoptotic signaling, we performed a TUNEL assay using fluorescence microscopy (Fig 2C). TUNEL-positive cells with fragmented DNA showed a green fluorescent signal in the DAPI-stained nuclei, indicating that DNA damage had occurred and that Dex-IR induced apoptosis in the H1650 cells.

### Dex-IR provokes cell cycle arrest

**Fig 3:**
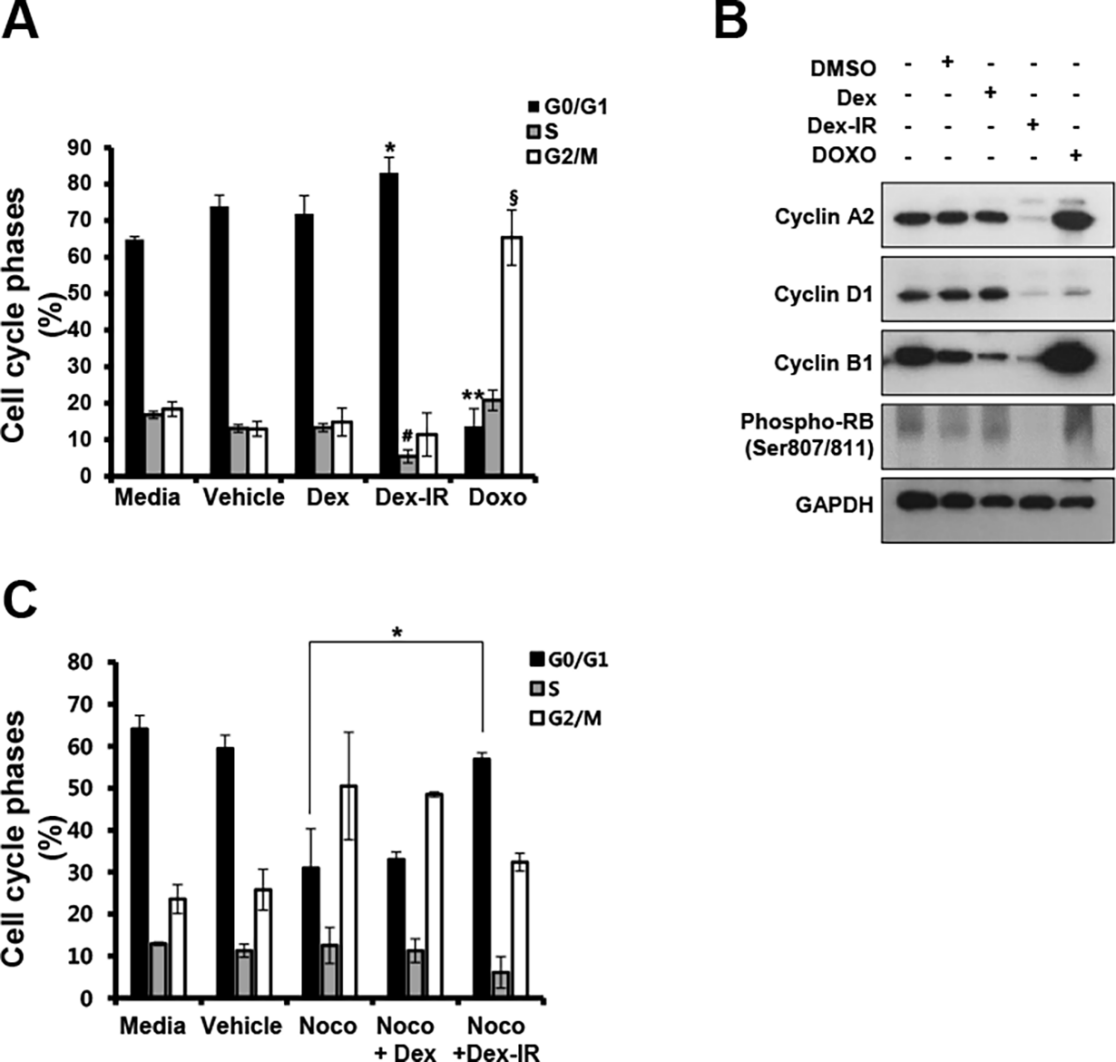
Dex-IR induced H1650 cell cycle arrest. (A) Cell cycle analysis following drug treatment for 72 h. The bar graph of the results of the cell cycle analysis of drug-treated H1650 cells shows the percentages of cells in different phases of the cell cycle (G_0_/G_1_, S, and G_2_/M). The bar graph shows the mean ± SEM of three independent experiments (*P < 0.05 *vs*. G_0_/G_1_ phase in the medium; ^#^*P* < 0.05 *vs*. S phase in the medium; and ^§^*P* < 0.05 *vs*. G2/M phase in the medium). (B) Cell lysates were analyzed by Immunoblot analysis using specific antibodies against the cyclins A2, D1, B1 and Phospho-Rb. Protein loading was normalized based on GAPDH. (C) Cells were treated with 400 nM nocodazole for 24 h, and then further treated with Dex or Dex-IR for 24 h. They were then harvested, and subjected to cell cycle analysis. The bar graph shows the mean ± SEM of three independent experiments (*P < 0.05 vs. G_0_/G_1_ phase in the Nocodazole).

Given the inhibitory effect of Dex-IR on the proliferation of lung cancer cells, we examined whether Dex-IR modulated the cell cycle. Cells were treated with Dex, Dex-IR, or DOXO for 72 h and stained with PI. The number of Dex-IR (100 ug/mL)-treated H1650 cells in G_0_/G_1_ phase increased from 64.8 to 83.1%, while the number in S phase decreased from 17.5 to 4.2% compared with control and Dex-treated cells (Fig 3A). However, a vast majority of cells (more than 60%) even in the control were arrested at the G_0_/G_1_ phase. To clearly determine whether Dex-IR induces this G_0_/G_1_ phase arrest, we used nocodazole (a mitotic inhibitor) to prime the cell at the G_2_/M phase of the cell cycle. Twenty-four hours after pretreatment with nocodazole, the cells were treated with Dex or Dex-IR for 24 h, and then harvested for cell cycle analysis. As seen in Fig 3C, co-treatment with Dex-IR strongly increased the number of cells in the G_0_/G_1_ phase and reduced the number of G_2_/M phase-arrested cells, when compared to the case for the nocodazole-treated cells (G_0_/G_1_; from 24.3 to 58%, G_2_/M; 65.2 to 39.8%). Collectively, these data suggest that Dex-IR mediated cell cycle arrest, predominantly inducing G_0_/G_1_ phase arrest in H1650 cells. To corroborate these observations, we examined the expression of proteins associated with the cell cycle using immunoblot analysis of lysates from H1650 cells treated with Dex, Dex-IR, or DOXO for 24 h and examined expression of the cyclins A2, B1, and D1. Dex-IR-treated H1650 cells showed decreased expression of the cyclins A2, B1 and D1 (Fig 3B). We also examined the effect of Dex-IR on phosphorylation of Rb at the CDK4/cyclinD1-specific site, and found that the expression level of phosphorylated-Rb was drastically reduced by Dex-IR, than by Dex. DOXO inhibits the degradation of cyclin B1, resulting in a G_2_M phase arrest as a result of cyclin B1 accumulation [17, 18]. These results suggested that Dex-IR is involved in the downregulation of cyclins, indicating that it led to cell cycle arrest at the G_0_/G_1_ phase, and ultimately inhibited the growth of lung cancer cells.

### Dex-IR inhibits H1650 cell invasion by regulating MMP9 activity

**Fig 4:**
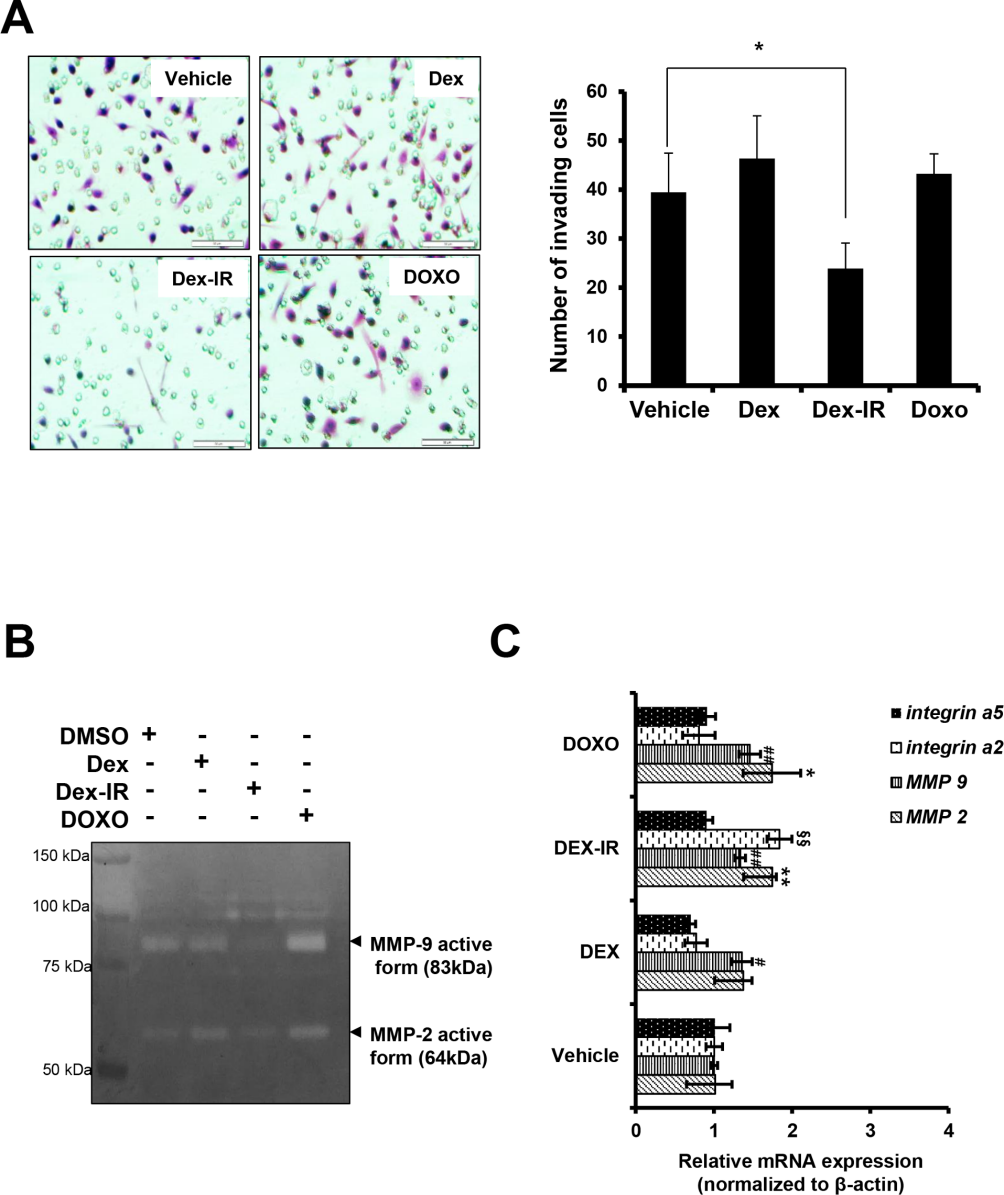
Dex-IR inhibits the invasiveness of H1650 cells. (A) Invasion assay of H1650 cells treated with the drugs as described in Fig 1. The Transwell invasion assay showed that Dex-IR suppressed the invasion of H1650 cells. Images were captured at a magnification of 400× (A, left panel). Scale bars, 100 μm. Graphical representation of the number of invasive H1650 cells per microscopic field. Each column and bar shows the SEM from three independent experiments (*P < 0.05 *vs*. vehicle) (A, right panel). (B) Inhibition of MMP9 activity in conditioned medium from H1650 cells treated with Dex-IR at the indicated concentration and incubated for 18 h was evaluated using gelatin zymography. Representative data from a single experiment are shown. The left lanes are standard markers. (C) qRT-PCR analysis of the MMP2, MMP9, integrin α2, and integrin α5 gene expression in cells 6 h after treatment with drugs. Each experiment was repeated three times and the results shown are representative of the three independent experiments. The bar graph shows the mean ± SEM of three independent experiments (*P < 0.05 vs. MMP2 expression in vehicle; ^#^P < 0.05 vs. MMP9 expression in vehicle; ^§^P < 0.05 vs. integrin α2 expression in vehicle).

Based on our observation that Dex-IR induced morphological changes in H1650 cells and increased the number of floating cells (Fig 1C), we explored the inhibitory effects of Dex-IR on cell invasion. H1650 cells were treated with Dex, Dex-IR, or DOXO as a chemoattractant in the lower part of Transwells for 24 h. The invasion assay showed that Dex-IR significantly suppressed tumor cell invasion compared with vehicle control cells (Fig 4A). We further examined the matrix metalloproteinase (MMP) activity of the Dex-IR-treated H1650 cells using zymography. This revealed that MMP9 was markedly decreased in Dex-IR-treated cells compared with the vehicle control (Fig 4B). To verify whether Dex-IR inhibited the expression of genes associated with invasion or adhesion, quantitative RT-PCR was performed using Dex-IR-treated H1650 cells. This revealed that Dex-IR did not decrease the levels of transcription of MMP2, MMP9, integrin α2, and integrin α5 compared with Dex. These observations imply that Dex-IR can reduce the invasion ability of H1650 cells by suppressing MMP9 activity.

## Discussion

Dexamethasone is used in the treatment of many diseases, including autoimmune disorders and cancers, despite its many side effects. The anticancer effects of Dex in the treatment of solid cancers have been reported in recent years [3–6, 19, 20]. Nevertheless, the mechanism by which Dex inhibits tumor cell growth remains controversial. In this study, we found that γ-irradiated Dex (Dex-IR) exhibited anticancer activity and reduced the viability and invasiveness of NSCLC cells.

We modified Dex with ionizing radiation and developed a potential anticancer candidate for lung cancer cells that showed better anticancer potency than the parent molecule, Dex. The ionizing radiation produced remarkable changes in the chemical properties of Dex. These changes caused the production of degradation products, such as methanol vapor and carbon monoxide from hydroxyl and carbonyl groups, as confirmed by LC-MS analysis. In addition, the main peak of Dex disappeared following γ-irradiation, which occurred simultaneously with the detection of five novel peaks.

In the present study, although we investigated the anticancer activity on NSCLC cells using mixtures of the dexamethasone derivatives, we believe that a potential possibility of these derivatives playing an important role in the inhibition of NSCLC was revealed. Even though all of the dexamethasone derivatives were not yet isolated, we succeeded in separating the three different fractions from mixture of derivatives. In order to evaluate the anticancer effect of Dexamethasone derivatives, we carried out the MTT assay to determine their cytotoxic effects on NSCLC. Only one of these fractions inhibited the proliferation of NSCLC cells, similar to Dex-IR at a concentration of 100 μg/ml (S1 Fig). The studies about the individual compounds of these dexamethasone derivatives will constitute our further investigations. Although further detailed molecular-based studies about the anticancer effects of dexamethasone derivatives created by the gamma irradiation of dexamethasone on lung cancer cells are yet to be determined, our results strongly suggest that there is a direct link between the chemical derivatives of dexamethasone and inhibition of NSCLC cell growth.

Ionizing radiation has been used because it allows for the introduction of energy into materials to discover favorable changes. Irradiated materials with sufficiently high energy can decompose to yield very reactive intermediate molecules, and form new ones. These changes could be applied to make the materials useful for advanced technologies. However, reproducibility is an important issue for the application of radiation-induced chemical reactions. We have found that the reproducibility of radiolytic dexamethasone decomposition was represented when dexamethasone solution with different initial concentrations (1 mg/ml or 1 g/L) was irradiated by ionizing radiation at a total dose of 20 kGy. This indicated that gamma irradiation was an effective way of producing improved innovative dexamethasone derivatives, which gave us a new insight into developing effective novel drugs.

Our results show that Dex-IR-induced apoptosis is caspase-3 dependent (Fig 2B). However, DMSO exposure also caused the activation of caspase-3. We used 1% DMSO (v/v) as a vehicle control due to the solubility of the drugs at this concentration. Usually, DMSO does not have toxic effects on cells at a concentration of 0.1% (v/v). However, at DMSO concentrations > 1%, cytotoxic effects have been reported [21]. Thus, it is assumed that activation of caspase-3 by 1% DMSO (v/v) due to the low-dose toxicity of DMSO itself is unexpected. Although DMSO-induced cytotoxicity was observed, caspase 3 activity by Dex-IR increased further, when compared to the DMSO-treated group, which was a solvent control. This result suggests that Dex-IR induces apoptosis through caspase activation.

In this study, we demonstrated that treatment with Dex-IR markedly inhibited lung cancer cell proliferation. Dex-IR also significantly suppressed the viability of NSCLC cells and induced apoptotic cell death via caspase/PARP-dependent pathways. In addition, treatment with Dex-IR induced phospho-RB and cyclin A2/D1/B2-dependent cell cycle arrest. Moreover, Dex-IR significantly inhibited the invasiveness of lung cancer cells by modulating the suppression of MMP9 activity. In conclusion, our results provide the first evidence that γ-irradiated Dex represents a novel class of anticancer agents for the treatment of lung cancer. Although the compounds present in Dex-IR remain to be identified, such modification could enhance pharmacological properties and anticancer activities of glucocorticoids.

## Supporting information

S1 Fig Inhibition of cell proliferation induced by single fraction isolated from Dex-IR. H1650 cells were treated with vehicle (0.2% MeOH), S1(single faction 1), S2(single fraction 2), S3(single fraction 3), Dex and Dex-IR at a concentration of 100 ug/ml for 24 h. The cell proliferation was assessed by MTT assay. The bar graph shows the mean ± SEM from three independent experiments (**, P < 0.01; ***, P < 0.001 *vs*. Media).
(PDF)
