# Supplementary material for: Dexamethasone modified by gamma-irradiation as a novel anticancer drug in human non-small cell lung cancer
Source: PLoS One. 2018 Apr 4;13(4):e0194341. doi: 10.1371/journal.pone.0194341 (PMC5884514; doi:10.1371/journal.pone.0194341)
Supplement: S1 Fig — H1650 cells were treated with vehicle (0.2% MeOH), S1(single faction 1), S2(single fraction 2), S3(single fraction 3), Dex and Dex-IR at a concentration of 100 ug/ml for 24 h. The cell proliferation was assessed by MTT assay. The bar graph shows the mean ± SEM from three independent experiments (**, P < 0.01; ***, P < 0.001 vs. Media). (PDF) [file pone.0194341.s001.pdf]

Supplementary Figures

Dexamethasone modified by gamma-irradiation as a novel anticancer drug in human non-small cell lung cancer

Eun-Hee Lee, Chul Hong Park, Hyo Jin Choi, Remigius Ambrose Kawala, Hyoung-Woo Bai and Byung Yeoup Chung

Contents:

S1 Fig. Inhibition of cell proliferation induced by single fraction isolated from Dex-IR

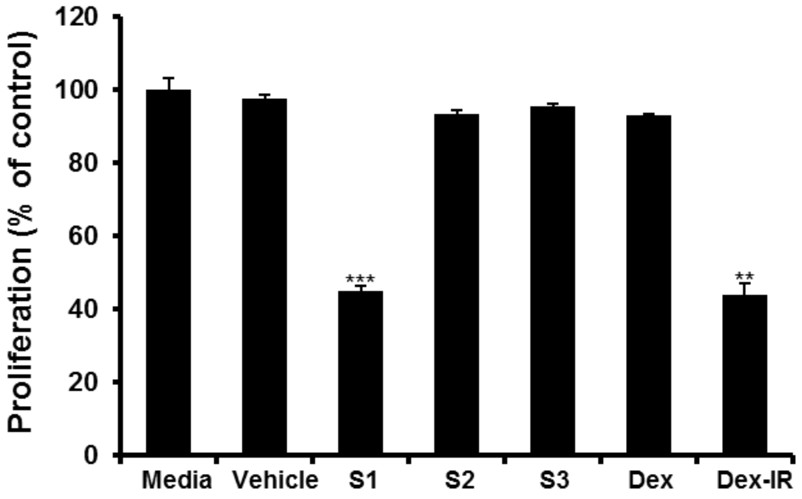

S1 Fig. Inhibition of cell proliferation induced by single fraction isolated from Dex-IR

H1650 cells were treated with vehicle (0.2% MeOH), S1(single faction 1), S2(single fraction 2), S3(single fraction 3), Dex and Dex-IR at a concentration of 100 ug/ml for 24 h. The cell proliferation was assessed by MTT assay. The bar graph shows the mean ± SEM from three independent experiments (**, P < 0.01; ***, P < 0.001 *vs*. Media).
